# Highly Efficient Near Infrared Photothermal Conversion Properties of Reduced Tungsten Oxide/Polyurethane Nanocomposites

**DOI:** 10.3390/nano7070191

**Published:** 2017-07-22

**Authors:** Tolesa Fita Chala, Chang-Mou Wu, Min-Hui Chou, Molla Bahiru Gebeyehu, Kuo-Bing Cheng

**Affiliations:** 1 Department of Materials Science and Engineering, National Taiwan University of Science and Technology, Taipei 10607, Taiwan, R.O.C; tolesafita@gmail.com (T.F.C.); bear200718@gmail.com (M.-H.C.); ycom5647@gmail.com (M.B.G.); 2 Department of Fiber and Composite Materials, Feng Chia University, Taichung 40724, Taiwan, R.O.C; kbcheng@fcu.edu

**Keywords:** nanocomposites, tungsten trioxide, photothermal conversion, polyurethane, near infrared ray

## Abstract

In this work, novel WO_3-*x*_/polyurethane (PU) nanocomposites were prepared by ball milling followed by stirring using a planetary mixer/de-aerator. The effects of phase transformation (WO_3_ → WO_2.8_ → WO_2.72_) and different weight fractions of tungsten oxide on the optical performance, photothermal conversion, and thermal properties of the prepared nanocomposites were examined. It was found that the nanocomposites exhibited strong photoabsorption in the entire near-infrared (NIR) region of 780–2500 nm and excellent photothermal conversion properties. This is because the particle size of WO_3-*x*_ was greatly reduced by ball milling and they were well-dispersed in the polyurethane matrix. The higher concentration of oxygen vacancies in WO_3-*x*_ contribute to the efficient absorption of NIR light and its conversion into thermal energy. In particular, WO_2.72_/PU nanocomposites showed strong NIR light absorption of ca. 92%, high photothermal conversion, and better thermal conductivity and absorptivity than other WO_3_/PU nanocomposites. Furthermore, when the nanocomposite with 7 wt % concentration of WO_2.72_ nanoparticles was irradiated with infrared light, the temperature of the nanocomposite increased rapidly and stabilized at 120 °C after 5 min. This temperature is 52 °C higher than that achieved by pure PU. These nanocomposites are suitable functional materials for solar collectors, smart coatings, and energy-saving applications.

## 1. Introduction

The development of nanomaterials capable of effectively absorbing near-infrared (NIR) radiation with a broad working waveband has increasingly attracted attention from the viewpoint of energy economization for applications in photothermal therapy, solar collectors, smart windows, and optical filters [1,2,3]. Solar energy is the most promising sustainable energy source among the existing sources of renewable energy and it can be converted to thermal energy by using solar collectors. The efficiency of conversion of solar energy into heat energy is mainly determined by the optical properties of the surface of the absorber, which should show strong absorption of solar radiation [4,5,6,7]. Photothermal materials convert light to heat and are widely used as absorbers. The process of converting light to heat involves absorption of photon energy of a specific wavelength by the photothermal material followed by conversion into thermal energy under optical illumination [8,9]. In this regard, plasmonic nanoparticles have received considerable attention in the past decade because of their novel and tunable localized surface plasmon resonance in the visible and NIR regions [10]. Several plasmonic nanomaterials with NIR photothermal conversion properties have been studied in the field of biological medicine, especially in photothermal therapy. Typical examples include carbon-based materials such as carbon nanotubes [11], graphene, and reduced graphene oxide [12,13],which exhibit relatively low absorption coefficients in the NIR region, and noble metal nanostructures including Pd-based nanosheets [14], gold nanorods [11,15], gold nanoshells [16,17], and gold nanocages [18]. However, all of the above mentioned materials can only absorb certain frequencies of NIR radiation and are not effective in the entire NIR wavelength range [19]. In addition, gold is an expensive noble metal and the preparation of its nanostructures with NIR photothermal conversion properties usually requires accurate synthesis conditions or depositions process which is relatively expensive and limit its further application [20]. Thus, there is a need to develop a low-cost and simple method for the preparation of NIR-absorbing nanomaterials for photothermal applications. 

Tungsten trioxide (WO_3_) has been recognized as one of the most promising semiconductor materials for gas sensors, photovoltaic organic solar cells, and electrochromic and photocatalytic applications owing to its suitable band gap (2.62 eV) and environmental benignity [21,22,23]. It has been reported that transition metal oxides are interesting candidates for photothermal applications as they exhibit localized surface plasmon resonance (LSPR) [24]. Particularly, non-stoichiometric tungsten oxide (WO_3-*x*_) nanocrystals are of significant interest because of their strong LSPR effect, which gives rise to strong photoabsorption peaks in the NIR region [25,26]. The strong NIR absorption properties of WO_3-*x*_ can be obtained by either reducing the oxygen content or adding ternary alkali metals [27,28,29]. Takeda and Adachi have reported the optical properties of reduced tungsten oxide under the H_2_/N_2_ gas atmosphere [28]. Recently, tungsten oxide-like monoclinic W_18_O_49_ (WO_2.72_) has attracted considerable attention for various applications, such as transparent smart windows, photocatalysts, and imaging guided photothermal therapy [30,31,32,33] because of its unusual defect structure and intense NIR photoabsorption. These properties motivated us to develop novel WO_3-*x*_/polyurethane (PU) nanocomposites for NIR photothermal conversion applications. PU is an attractive material and one of the most actively investigated polymers because of its outstanding properties, such as thermal and chemical stabilities, high impact strength, and easy processing [34,35]. Due to these advancements, PU has been widely applied in many fields, such as breathable waterproof textiles, functional coatings, paints, adhesives and foams, etc. [36,37,38,39]. The easy fabrication and low cost of PU composites is highly desirable for practical applications. Therefore, PU was selected as the matrix and incorporated with WO_3-*x*_ nanoparticles to provide more functions. It is believed that the good dispersion states of nanoparticles in polyurethane matrix using ball milling significantly affects the NIR absorption and photothermal conversion properties of the nanocomposites.

In this work, reduced tungsten oxide (WO_3-*x*_) nanoparticles were first prepared from pure WO_3_ by reduction in a tube furnace under a carbon monoxide atmosphere [40]. The WO_3-*x*_ nanoparticles were mixed with PU in a dimethylformamide (DMF) solution and then stirred by ball milling, followed by continuous stirring using a planetary mixer/de-aerator. Polyurethane was used as the matrix for WO_3-*x*_ nanoparticles because good dispersion states of the particles were achieved by ball milling them together. The dispersion state of a nanocomposite has a significant effect on its NIR absorption and photothermal conversion properties. Thermal properties of the resulting nanocomposites such as conductivity, absorptivity, and resistivity were investigated. In addition, the effects of phase transformations and weight fractions of reduced tungsten oxide on the photothermal performance and the thermal properties of the nanocomposites were studied.

## 2. Results and Discussion

### 2.1. Characterization of WO_3-x_ Nanoparticles

Reduced tungsten oxide was prepared from pure tungsten trioxide via reduction under an atmosphere of carbon monoxide in a tube furnace. The reduction of mechanisms of tungsten oxides by CO could be expected to proceed as follows:

(1) $${\mathrm{WO}}_{x}\left(s\right)+\mathrm{CO}\left(g\right)↔{\mathrm{WO}}_{y}\left(s\right)+{\mathrm{CO}}_{2}\left(g\right),\left(\mathrm{where}\;x>y\right),$$

**Figure 1:**
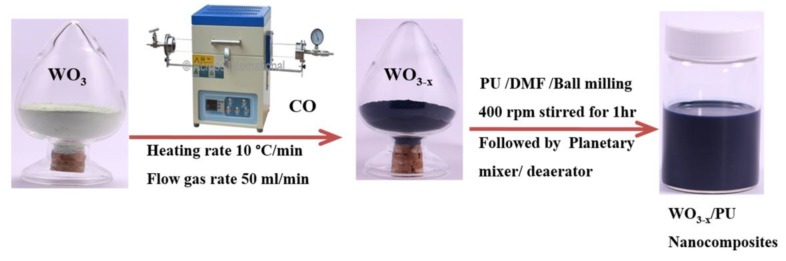
Schematic illustrations of the preparation of WO_3-*x*_ and WO_3-*x*_/PU nanocomposites.

The overall procedure for the preparation of WO_3-*x*_ and its nanocomposites (WO_3-*x*_/PU) is schematically depicted in Figure 1.

**Figure 2:**
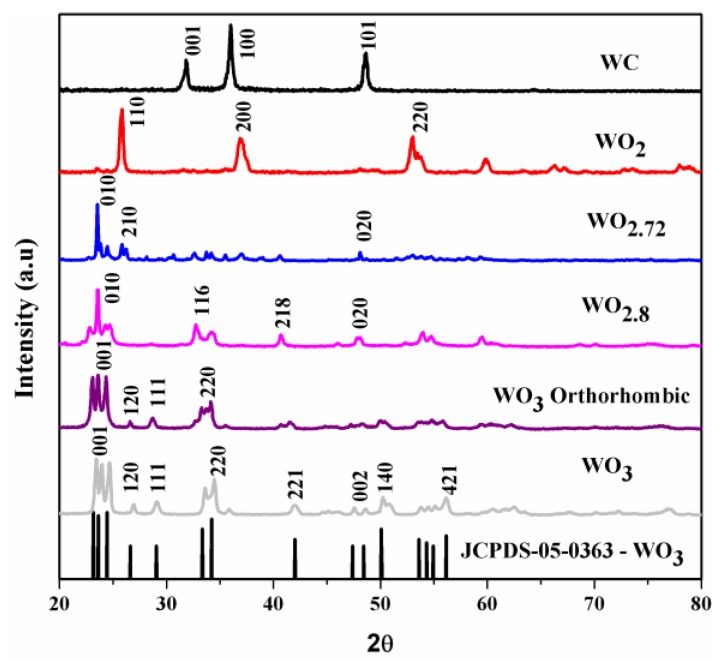
X-ray diffraction (XRD) patterns of pure tungsten trioxide before reduction and its sub-oxides after reduction under CO atmosphere.

**Figure 3:**
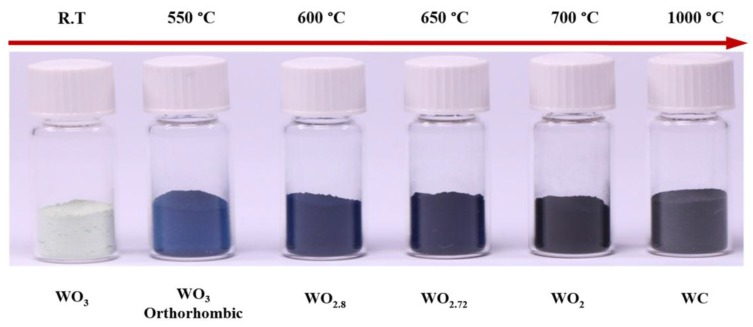
Variation in the color of reduced tungsten oxide powder prepared by reduction at different temperatures under CO atmosphere.

Figure 2 shows the typical X-ray diffraction (XRD) patterns of pure WO_3_ before reduction and its sub-oxides after reduction. The WO_3-*x*_ phase undergoes phase transformations during reduction, which affects its stoichiometry. As shown in Figure 2, multiple peaks were observed in the XRD pattern of pure WO_3_. At 550 °C thermal treatment, WO_3_ exhibited an orthorhombic crystal structure (JCPDS-05-0364). However, increasing reduction temperature the multiple diffraction peaks of pure WO_3_ at 23.3° became sharper and narrower, indicating the phase transformation of WO_3_ nanoparticles. The intermediate phases WO_2.8_ (JCPDS-05-0386), WO_2.72_ (JCPDS-05-0392), and WO_2_ (JCPDS-02-0414) were obtained at reduction temperatures of 600, 650, and 700 °C, respectively. A further increase in the reduction temperature to 1000 °C resulted in the formation of WC particles (JCPDS-02-1055). These results match well with those described in previous reports wherein the final product of reduction that was formed under an atmosphere of hydrogen and carbon was tungsten (W) [41,42]. In addition, based on the experimental data, the diffraction peaks of reduced tungsten oxide at 650 °C indicated the formation of a monoclinic phase. The interplanar spacing of 0.37 nm determined from the XRD pattern corresponded to that of the (010) plane of the monoclinic crystal structure of WO_2.72_ (W_18_O_49_). Figure 3 shows the variation in the color of the WO_3-*x*_ powder obtained after reduction at different temperatures under CO atmosphere. The color changed from yellow for WO_3_ to dark blue for WO_3-*x*_ and black for WC.

**Figure 4:**
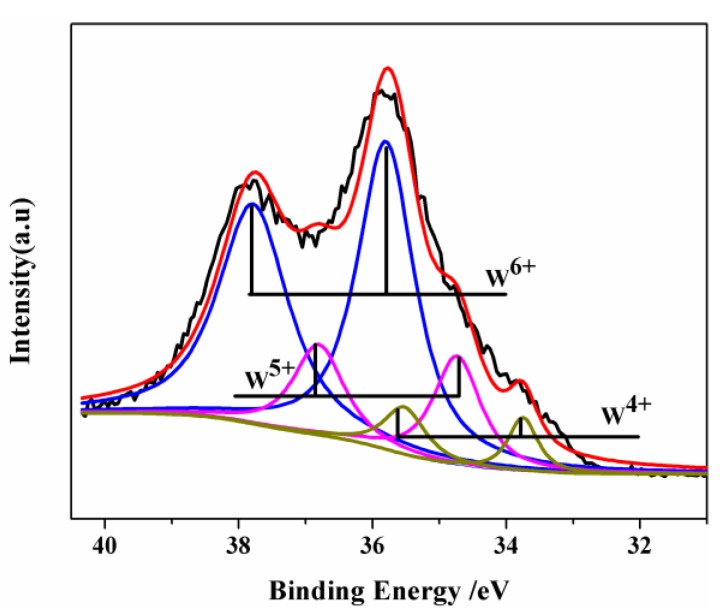
W4f X-ray photoelectron spectroscopy (XPS) spectra of WO_2.72_ prepared by reduction at 650 °C under CO atmosphere.

The chemical composition and the valence states of the prepared nanoparticles were examined by X-ray photoelectron spectroscopy (XPS). A complex energy distribution of W4f (where W is tungsten atoms, 4 is principal quantum number and f is core or inner atomic orbital) photoelectrons was obtained, as shown in Figure 4. The W4f core-level spectrum was fitted to three spin-orbit doublets corresponding to the three different oxidation states of W atoms. The W4f_5/2_ and W4f_7/2_ peaks at 37.86 and 35.77 eV, respectively, can be attributed to the +6 oxidation state of the W atoms. The second doublet at lower binding energy values of 34.8 and 36.9 eV arises due to the emissions from W4f_7/2_ and W4f_5/2_ core levels, respectively, corresponding to the +5 oxidation state of W. The third doublet observed at 33.8 and 35.75 eV corresponds to the tungsten +4 oxidation state. These three oxidation states are typically found in WO_2.72_ nanomaterials [43,44].

**Figure 5:**
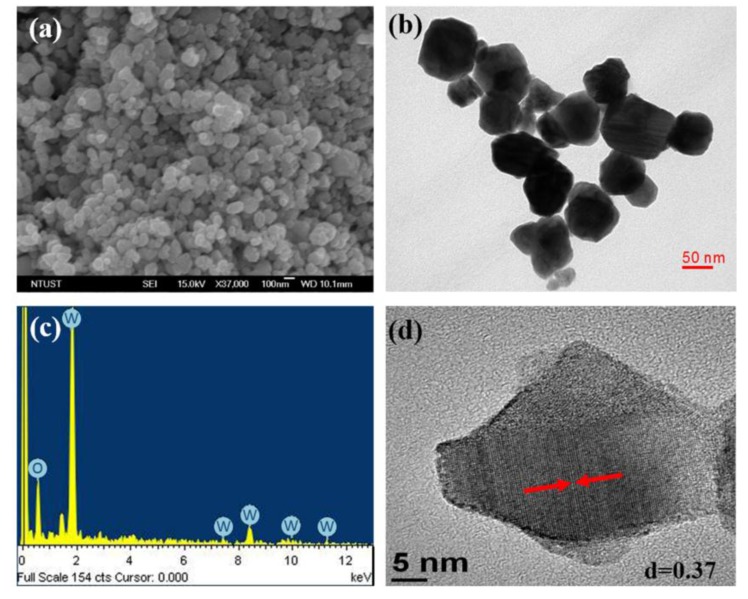
(**a**) Field Emission Scanning Electron Microscopy (FESEM); (**b**) Transmission electron microscopy (TEM); (**c**) Energy dispersive X-ray analysis (EDX) spectrum; and (**d**) High-Resolution Transmission Electron Microscopy (HRTEM) images of WO_2.72_ powder.

The Field Emission Scanning Electron Microscopy (FESEM) image of the as-prepared WO_2.72_ powder is shown in Figure 5. It is evident from Figure 5a that the sample consists of spherical particles with a relatively uniform size ranging from 57 to 106 nm. The morphologies and microstructures of the powders were further investigated by Transmission electron microscopy (TEM) and High-Resolution Transmission Electron Microscopy (HRTEM) analyses. The TEM analysis confirmed the formation of nanoparticles with an average particle size of 78 nm (Figure 5b), which was consistent with the particle size determined from the SEM analysis. The energy dispersive X-ray analysis (EDX) spectrum shown in Figure 5c confirmed the presence of W and O elements in the sample; the peaks corresponding to Cu originate from the copper grid substrate which was used for the TEM measurements. The spacing between adjacent lattice planes was found to be 0.37 nm from the HRTEM image (Figure 5d). This spacing corresponds to the (010) plane of monoclinic WO_2.72_ phase, which is consistent with the results of XRD analysis.

### 2.2. Optical Properties and Morphologies of WO_3-x_/PU Nanocomposites

**Figure 6:**
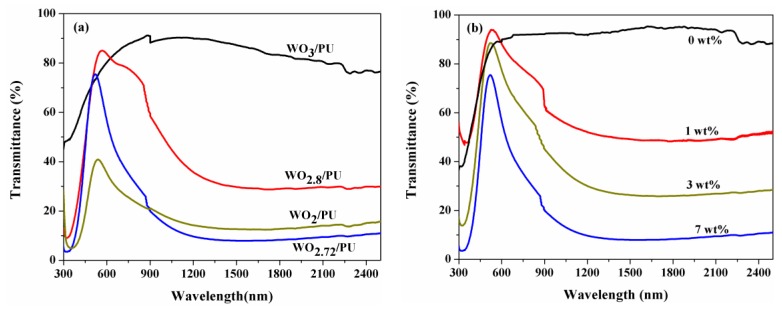
UV-Vis-NIR transmittance spectra of (**a**) WO_3_/PU, WO_2.8_/PU, and WO_2.72_/PU nanocomposites prepared with tungsten oxide weight fraction of 7 wt %; and (**b**) WO_2.72_/PU nanocomposites prepared with different weight fractions of WO_2.72_ (0–7 wt %).

The optical properties of the prepared nanocomposites were evaluated by using a UV-Vis-NIR spectrophotometer in the range of 300–2500 nm. The homogeneous sample solution prepared by the stirred ball milling method was spin-coated on quartz glass substrates at 800–1500 rpm. Figure 6a shows the UV-Vis-NIR transmittance spectra of the nanocomposites prepared with the same weight fraction (7 wt %) of reduced tungsten oxide. The transmittance values of WO_2.8_/PU and WO_2.72_/PU nanocomposites in the visible region (400–780 nm) were ca. 85.6 and 75%, respectively. The transmittance of the WO_2.72_/PU nanocomposites was very low (8%) in the range of 780–2500 nm, which suggested that the WO_2.72_/PU nanocomposites exhibit stronger NIR absorption (ca. 92%) compared to other nanocomposites. This is because the absorption of NIR radiation is closely related to the presence of free electrons or oxygen-deficiency-induced small polarons formed during the reduction process [45]. For comparison, the transmittance spectra of pure PU and WO_3_/PU nanocomposites were also recorded. It was found that these nanocomposites exhibited high transmittance (>85%) in the entire UV-Vis-NIR region (300–2500 nm), indicating negligible photoabsorption in the NIR region. The WO_2_/PU nanocomposites exhibited very low transmittance in the visible region in comparison with the WO_2.8_/PU nanocomposites and a lower absorption in the NIR region compared to the WO_2.72_/PU nanocomposites. This behavior may be attributed to the excessive reduction of WO_2_ and consequently the lower number of free electrons or polarons that are formed [28]. The strong NIR photoabsorption of the WO_3-*x*_/PU nanocomposites is attributed to the presence of WO_3-*x*_ nanoparticles that efficiently absorb NIR radiation and convert it to thermal energy via the strong localized surface plasma resonance effect [25,46]. Thus, it is necessary to evaluate the effect of different weight fractions of WO_2.72_ on the optical properties of WO_2.72_/PU nanocomposites.

Figure 6b shows the UV-Vis-NIR-transmittance spectra of WO_2.72_/PU nanocomposites prepared with different weight fractions of WO_2.72_. According to the experimental results, as the weight fraction of WO_2.72_ was increased from 0 to 7 wt %, the NIR transmittance of the nanocomposites decreased, indicating that the absorption of NIR radiation had significantly increased. This implies that the amount of nano-sized WO_2.72_/PU required for efficient absorption of NIR radiation increases with increase in the content of reduced tungsten oxide (WO_2.72_). The strong absorption of WO_3-*x*_/PU nanocomposites in the NIR region motivated us to further study their morphologies and photothermal conversion properties.

**Figure 7:**
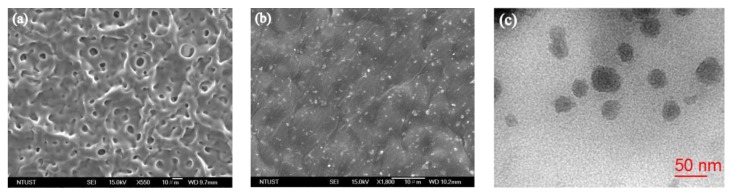
FESEM images of (**a**) pure polyurethane (PU); (**b**) WO_2.72_/PU prepared with 7 wt % of WO_2.72_; and (**c**) TEM image of WO_2.72_/PU nanocomposites prepared with 7 wt % of WO_2.72_.

The physical properties and efficiency of inorganic-organic composites can generally be enhanced by dispersing the inorganic filler in a polymer matrix [47]. The morphologies and dispersion states of reduced tungsten oxide nanoparticles in the PU matrix were investigated by FESEM and TEM techniques. Figure 7a,b show the typical FESEM images of pure PU and WO_2.72_/PU nanocomposites prepared with 7 wt % of WO_2.72_. While the pure PU sample exhibited a porous and rough surface, whereas the surface of WO_2.72_/PU nanocomposites showed the presence of white spots owing to the incorporation of WO_2.72_ nanoparticles in PU. In addition, the surface of the nanocomposite samples was uniform and smooth, and no cracks were observed. The TEM images also revealed that the WO_2.72_ nanoparticles were well-dispersed in the PU matrix, and nanocomposites with particle sizes ranging from 20 to 40 nm (Figure 7c) were formed. The particle sizes of WO_2.72_ in the PU matrix decreased significantly as a result of the grinding process. Ball-milling ground the materials to powder with shear stress and reduced the average particle sizes effectively, thus increasing the performance of these nanocomposites. However, the detail study of about the effects size, morphology of WO_2.72_ before and after ball milling on NIR absorption properties of nanocomposites will be our next work.

### 2.3. NIR Photothermal Conversion and Thermal Properties of Nanocomposites

**Figure 8:**
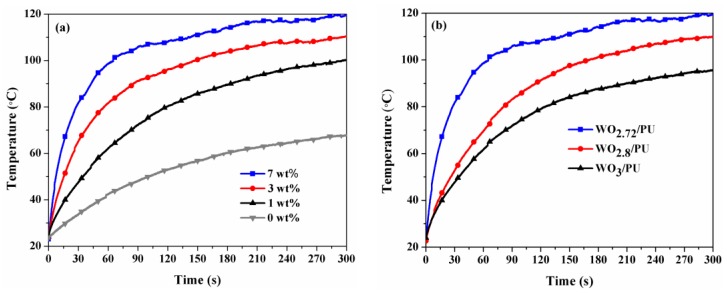
Temperature distribution of (**a**) WO_2.72_/PU nanocomposites at different weight fractions (0–7 wt %) of WO_2.72_ and (**b**) WO_3_/PU, WO_2.8_/PU, and WO_2.72_/PU with 7 wt % as a function of time under infrared light irradiation.

Figure 8a shows the effect of different weight fractions of WO_2.72_ nanoparticles on the photothermal conversion characteristics of the corresponding nanocomposites. The results showed that the temperature of the nanocomposites increases rapidly with increase in the weight fraction of WO_2.72_ from 0 to 7 wt %. The temperature changes (Δ*T*) were 44.8, 77, 87.5, and 96.5 °C for WO_2.72_/PU nanocomposites prepared with 0, 1, 3, and 7 wt % WO_2.72_, respectively, after light irradiation for 300 s. It is worth noting that the temperature for the 7 wt % sample increased rapidly and stabilized at 120 °C, which is 52 °C higher than the temperature attained by pure PU. For comparison, the NIR photothermal conversion properties of WO_2.8_/PU and WO_3_/PU nanocomposites were also examined at the same weight fraction of 7 wt % under identical conditions. These results are shown in Figure 8b. The temperature of the WO_2.72_/PU nanocomposites increased rapidly to reach Δ*T* = 32.5 °C after 10 s and Δ*T* = 58.9 °C after 30 s, and gradually stabilized after 300 s. However, the Δ*T* for WO_2.8_/PU, WO_3_/PU, and pure PU were 41.9, 30.9, and 9.9 °C after 30 s, and stabilized at 86.6, 75.9, and 44.8 °C after 300 s, respectively. These results suggest that the WO_2.72_/PU nanocomposites exhibit faster photothermal conversion rate than WO_2.8_/PU, WO_3_/PU, and pure PU. After an irradiation time of 30 s, the photothermal conversion rates were determined to be 108.20, 57.04, 44.43, and 19.94 °C min^−1^ for WO_2.72_/PU, WO_2.8_/PU, WO_3_/PU, and pure PU, respectively. These results indicate that the photothermal conversion characteristics improve significantly when the oxygen content of the nanocomposites is reduced. The reduced oxygen content is responsible for the introduction of free electrons into the crystal structure and the resultant strong NIR absorption. Generally, during the irradiation process, the temperature of the nanocomposites initially increases sharply and then shows a gradual increase with increasing irradiation time. The photothermal conversion rate becomes lower with the further increase in the temperature owing to faster heat loss at higher temperatures [45,48,49,50,51]. The uniform dispersion and decrease in particle size of WO_2.72_ powders in polyurethane matrix after grinding resulted in much higher photothermal conversion properties (56.5 °C after 10 s) under IR irradiation compared to those reported for WO_2.72_ (36.5 °C after 10 s) [30,52]. In addition WO_2.72_/PU nanocomposites also shows higher photothermal conversion performance than gold nanostars coated with polydopamine and graphene oxide modified PLA microcapsules containing gold nanoparticles, which the temperature increment was only 35–50 °C after 300 s [53,54]. The developed WO_2.72_/PU nanocomposites exhibit extremely high photothermal conversions and the temperature reaches 120 °C after 5 min. To the best of our knowledge, this is the highest temperature that has been reported in the literature to date. Water can be efficient evaporated at such high temperature and, thus, the WO_2.72_/PU nanocomposites show great potential applications in solar energy collectors, such as vapor power steam generators and others functional foams and coatings, such as warm/heat coatings, etc.

Table 1 shows the thermal properties of the nanocomposites prepared with different contents of WO_2.72_. The thermal conductivity and absorptivity of the nanocomposites was found to increase with increasing weight fraction of WO_2.72_. Thus, the highest thermal conductivity and thermal absorptivity of 97.10 mWm^−1^K^−1^ and 496.80 Ws^1/2^m^−2^K^−1^, respectively, was observed for the nanocomposite with 7 wt % of WO_2.72_. However, the thermal resistance of the nanocomposites decreased with increasing weight fraction of WO_2.72_. This is because thermal resistance (resistance to heat flow) is inversely proportional to thermal conductivity [55]. For comparison, the thermal properties of the WO_3_/PU and the WO_2.8_/PU nanocomposites were also studied under similar conditions at the same weight fraction of 7 wt %. These results are summarized in Table 2. It was found that the WO_2.72_/PU nanocomposites showed the highest values of thermal conductivity and thermal absorptivity when compared to those of WO_3_/PU nanocomposites owing to the presence of unusual oxygen defect structures. 

## 3. Materials and Methods

### 3.1. Materials

The tungsten trioxide slurry in water dispersion that was prepared by the ball milling process and had its particle size measured and confirmed as being typically 60–110 nm was obtained from Advanced Ceramics Nanotech Co. Ltd., Taipei, Taiwan. PU/DMF solution with 30 wt % solid content was purchased from Gabriel Advanced Materials Co. Ltd., Taipei, Taiwan.

### 3.2. Preparation of WO_3-x_ Nanoparticles

The homogeneous yellow dispersion of WO_3_ was separated by centrifugation and dried in an oven at 60 °C. Subsequently, the as-obtained WO_3_ powder was reduced in a temperature-programmed tubular furnace under a carbon monoxide atmosphere at a heating rate of 10 °C min^−1^ and a carrier gas flow rate of 50 mL min^−1^. The reduction of time of WO_3_ was hold for 30 min and conducted under non-isothermal conditions in the temperature range of 550–1000 °C.

### 3.3. Preparation of WO_3-x_/PU Nanocomposites

The WO_3-*x*_/PU nanocomposites were prepared by a stirred ball milling process. The ball mill used was a high-performance batch-type stirred bead mill, Pulverisette classic line (Utek International Co. Ltd., Idar-oberstein, Germany). Yttrium-stabilized zirconia (95% ZrO_2_, 5% Y_2_O_3_) stirred beads with a diameter of 5 mm were used. For the typical stirred bead milling process, various amounts (wt %) of the WO_3-*x*_ powder were added to the PU-containing DMF solution and then dispersed in the stirred ball mill at an agitation speed of 400 rpm for 1 h. After ball milling, the solution was continuously stirred using a planetary mixer/deaerator (Mazerustar KK-250S Satellite Motion Mixer, Osaka, Japan) for 6 min to enable uniform dispersion and avoid the formation of air bubbles. Finally, a well-dispersed solution of WO_3-*x*_/PU was obtained.

For the preparation of nanocomposite films, the resultant homogenized solution was casted onto a cleaned slide glass and dried at 60 °C in a vacuum oven to remove the solvent. The as-prepared nanocomposites with different contents of WO_2.72_ (0, 1, 3, and 7 wt %) could be easily peeled off from the glass slides. For comparison, nanocomposites WO_2.8_/PU and WO_3_/PU with 7 wt % tungsten oxide were also prepared under identical conditions.

### 3.4. Characterization

X-ray diffraction (XRD) measurements were recorded with a BrukerD2 phaser diffractometer (Karlsruhe, Germany) using a Cu Kα radiation source in the scan range of 20–80° (2θ) at a scan rate of 2° min^−1^ and step size of 0.02°. The morphologies and sizes of the prepared powder samples and nanocomposites were studied by field emission scanning electron microscopy (FESEM, JSM6500F, JEOL, Tokyo, Japan) and transmission electron microscopy (TEM, JEOLJEM-2010, Tokyo, Japan). The surface compositions of the samples and the binding energies of the W4f core levels were determined by X-ray photoelectron spectroscopy (XPS, Perkin-Elmer PHI 5600, Waltham, MA, USA). The optical response of the coating was measured by using a spectrophotometer (JASCO V-670, Keith Link Technology, Jasco Analytical Instruments, Easton, MD, USA), which provided the transmittance in the UV, visible, and infrared ranges (300–2500 nm). In order to evaluate the photothermal conversion properties of the nanocomposites, the samples were irradiated with an infrared lamp at a power of 150 W and the temperature distribution was recorded by using a thermal imaging camera (FLIR P384A3-20, CTCT, Co. Ltd., Taipei, Taiwan). The thermal properties of the nanocomposites such as conductivity, absorptivity, and resistivity were measured by using the Alambeta instrument (Sensora Instruments, Thurmansbang, Germany).

## 4. Conclusions

In this work, WO_3-*x*_ nanoparticles were prepared from pure WO_3_ via thermal reduction and, subsequently, novel WO_3-*x*_/PU nanocomposites were prepared using the WO_3-*x*_ nanoparticles and PU by a simple stirred ball milling method. The particle size of the as-prepared nanocomposites was significantly reduced after ball milling. In addition, the FESEM, TEM, and UV-Vis-NIR absorption spectral analyses of the nanocomposites confirmed that the WO_3-*x*_ nanoparticles were well-dispersed in the PU matrix. The WO_3-*x*_ nanoparticles showed strong absorption of NIR light and rapid NIR photothermal conversion characteristics in the PU matrix. Among the different reduced tungsten oxide nanocomposites prepared in this work, WO_2.72_/PU with 7 wt % WO_2.72_ exhibited strong NIR light absorption, high thermal conductivity, high thermal absorptivity, and the highest photothermal conversion characteristics upon infrared light irradiation, owing to its unusual oxygen defect structure. The temperature change (Δ*T*) of the WO_2.72_/PU nanocomposites increased rapidly and reached 32.5 °C after 10 s and 58.9 °C after 30 s, before gradually stabilizing at 96.5 °C after 300 s under infrared light irradiation. In addition, the photothermal conversion rate of the WO_2.72_/PU nanocomposites was 108.20 °C min^−1^, which is very fast when compared to that of WO_2.8_/PU, WO_3_/PU, and pure PU after an irradiation time of 30 s. These results indicate a quick conversion of the absorbed NIR light energy to local heat energy on the WO_2.72_/PU nanocomposites.

## Figures and Tables

**Table 1:** Thermal properties of WO_2.72_/PU nanocomposites prepared with different weight fractions of WO_2.72_.

Weight Fractions of WO_2.72_ (wt %)	0	1	3	7
Thermal Conductivity (mWm^−1^K^−1^)	34.40	76.80	87.70	97.10
Thermal Absorption (Ws^1/2^m^−2^K^−1^)	211.37	446.80	467.43	496.80
Thermal Resistance (m^2^mkW^−1^)	11.40	9.50	7.75	7.2

**Table 2:** Thermal properties of WO_3_/PU, WO_2.8_/PU, and WO_2.72_/PU nanocomposites prepared with 7 wt % tungsten oxide.

Parameter	WO_3_/PU	WO_2.8_/PU	WO_2.72_/PU
Thermal Conductivity (mWm^−1^K^−1^)	37.20	68.40	97.10
Thermal Absorption (Ws^1/2^m^−2^K^−1^)	153.60	384.83	496.80
Thermal Resistance (m^2^mkW^−1^)	9.92	8.10	7.20
